# Revealing the Microstructure Evolution and Carbonation Hardening Mechanism of β-C_2_S Pastes by Backscattered Electron Images

**DOI:** 10.3390/ma12091561

**Published:** 2019-05-13

**Authors:** Songhui Liu, Xuemao Guan, Haibo Zhang, Yuli Wang, Mifeng Gou

**Affiliations:** School of Materials Science and Engineering, Henan Polytechnic University, 2001 Century Avenue, Jiaozuo 454000, China; liusonghuicbma@163.com (S.L.); guanxuemaohpu@sina.com (X.G.); goumifeng@hpu.edu.cn (M.G.)

**Keywords:** backscattered electron image, dicalcium silicate, carbonation, microstructure

## Abstract

β-dicalcium silicate (β-C_2_S) minerals were prepared. The compositions, microstructures, and distributions of the carbonation products of hardened β-C_2_S paste were revealed by X-ray diffraction (XRD), Fourier transform-infrared (FT-IR) spectroscopy, and backscattered electron (BSE) image analysis. The results show that a dense hardened paste of β-C_2_S can be obtained after 24 h of carbonation curing. The hardened pastes are composed of pores, silica gel, calcium carbonate, and unreacted dicalcium silicate, with relative volume fractions of 1.3%, 42.1%, 44.9%, and 11.7%, respectively. The unreacted dicalcium silicate is encapsulated with a silica gel rim, and the pores between the original dicalcium silicate particles are filled with calcium carbonate. The sufficient carbonation products that rapidly formed during the carbonation curing process, forming a dense microstructure, are responsible for the carbonation hardening of the β-C_2_S mineral.

## 1. Introduction

Portland cement is the most widely consumed cementitious material in the world. However, due to the high calcium minerals such as tricalcium silicate, the production of Portland cement is an energy-intensive process which also releases a high amount of CO_2_. Reducing the calcium oxide content in Portland cement and developing low-calcium cementitious materials which can partially replace Portland cement are hot topics [1,2,3,4]. Among them, the carbonation-hardened cementitious materials with low-calcium silicates (β-C_2_S, γ-C_2_S, C_3_S_2_, and CS) as the main minerals have significantly reduced CO_2_ emissions and achieve excellent mechanical performance by a short carbonation curing process, and thus are receiving extensive attention all over the world.

Numerous research efforts have focused on the carbonation hardening mechanical performances, reaction product compositions, and microstructures of calcium silicates [5,6,7,8]. The results show that amounts of CaCO_3_ crystals (including calcite, vaterite, and aragonite) and highly polymerized SiO_2_ gels are formed within 24 h of carbonation curing, which contributes to the excellent mechanical strength of hardened pastes (achieving 70–80 MPa after carbonation for 24 h) [7,9]. However, little research has been done on the distributions and relative volume fractions of the above two types of carbonation products (CaCO_3_ crystal and SiO_2_ gel), which are of great significance for revealing the carbonation reaction process and hardening mechanism of low-calcium silicate-based cementitious materials.

In recent years, backscattered electron image quantitative analysis technology has been widely used to reveal the microstructure, hydration degree, and porous structure of cement-based materials [10,11,12,13,14]. Since cement clinkers and hydration products have different grayscale features, the relative volume fractions of each product with different grayscale features can be obtained by analyzing the gray level distributions of backscattered electron images. The hardened calcium silicate pastes after carbonation are composed of unreacted C_2_S, CaCO_3_, and amorphous SiO_2_ gel formed during the carbonation reactions. The average atomic numbers of C_2_S, CaCO_3_, and SiO_2_ are 14.56, 12.56, and 10.81, respectively, showing obvious grayscale features [15]. Therefore, the distributions and relative volume fractions of C_2_S, CaCO_3_, and SiO_2_ can be well characterized by backscattering electron image analysis. However, there are still few related reports. 

In this work, β-dicalcium silicate (β-C_2_S) minerals were prepared. The compositions, microstructure distributions, and relative volume fractions of the carbonation reaction products of hardened β-C_2_S pastes were determined by X-ray diffraction (XRD), Fourier transform-infrared spectroscopy (FT-IR), and backscattered electron (BSE) image analysis. Moreover, the microstructure formation process and carbonation hardening mechanism of β-C_2_S were also revealed.

## 2. Materials and Methods

### 2.1. Preparation of β-C_2_S

**Figure 1:**
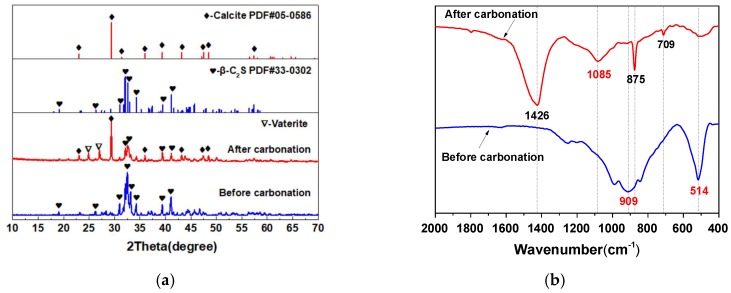
X-ray diffraction and Fourier transform-infrared spectra of β-C_2_S before and after carbonation. (**a**) XRD; (**b**) FT-IR.

76.9% of CaCO_3_ and 23.1% of SiO_2_ analytical purification reagents were weighed and mixed uniformly. Additionally, barium oxide with a mass fraction of 0.3% was added to the above mixture to prevent the conversion of β-C_2_S to γ-C_2_S. Then, the mixture was pelleted and calcined at 1350 °C for 2 h in a high-temperature furnace. Subsequently, the resulting clinker was quenched and re-calcined until the free-CaO content in the resulting clinker was negligible. Finally, the resulting β-C_2_S was ground to powder with a Blaine specific surface area of 4100 cm^2^/g. The polymorph of prepared C_2_S is β-C_2_S, which was determined by XRD [16]. The XRD pattern is shown in Figure 1.

### 2.2. Carbonation of β-C_2_S Pastes

The prepared β-C_2_S powder was mixed uniformly with 10% by mass of water. The wet mixture was then poured into a mold and subjected to a compression molding process under a molding pressure of 4 MPa at maximum pressure for 30 s. The obtained block size was about 40 × 40 × 50 mm. After demolding, the obtained block was immediately placed into a sealed carbonation reactor with a CO_2_ gas concentration of 99% and a CO_2_ pressure of 0.1 MPa at room temperature [17]. The sample was removed from the carbonation reactor after carbonation for 24 h.

### 2.3. Test Methods

The hardened pastes were crushed, dried, and ground with an agate mortar. The XRD and FT-IR spectra of the powder samples were determined to characterize the phase change before and after carbonation. The XRD patterns were obtained by using a Rigaku SmartLab diffractometer (Tokyo, Japan) with Cu K_α_ radiation (λ = 1.5406 Å) at the range of 10–70°. The FT-IR spectroscopy data were collected by using a Bruker V70 spectrometer (Billerica, MA, USA) at a range of 400–2000 cm^−1^ with a resolution of 4 cm^−1^. A small cut portion of the hardened pastes after carbonation was dried and epoxy impregnated. After impregnation, one of the surfaces was polished to 0.5 μm finish [13]. The polished surface was sputter-coated with a thin layer of gold (Au) and examined under a scanning electron microscope (SEM) in backscattered mode. A Merlin Compact ultrahigh-resolution field emission scanning electron microscope (SEM, Oberkochen, Germany) coupled with Oxford energy-dispersive spectroscopy (EDS, Abingdon, UK) at 20 kV was used to acquire the images. The gray level distribution analysis and phase separation of the acquired BSE images were performed by Image-Pro plus image analysis software. In order to ensure data consistency, each BSE image was acquired under the same test conditions (acceleration voltage, beam spot value, brightness, contrast, etc.), and all BSE images were subjected to the same image analysis step [18]. Each quantitative analysis result presented in this paper is an average of 10 BSE image analysis data, and the standard deviations are presented in brackets. 

## 3. Results and Discussion

### 3.1. The Carbonation Products of β-C_2_S Pastes

The XRD patterns of β-C_2_S minerals before and after carbonation are shown in Figure 1a. It was observed from Figure 1a that the diffraction patterns of the prepared β-C_2_S minerals match well with the β-C_2_S mineral PDF standard card. After carbonation curing, the diffraction peak intensities of β-C_2_S minerals decreased significantly, and the diffraction peaks of calcite and vaterite appeared. Moreover, the diffraction peak intensity of calcite was significantly higher than that of vaterite, indicating that a large amount of CaCO_3_ crystals dominated by calcite were formed during the carbonation curing process. There was no diffraction peak of SiO_2_ after carbonation, and SiO_2_ exists in the form of an amorphous gel. 

In order to further verify the structure of the SiO_2_ gel formed during the carbonation, the FT-IR spectra of the β-C_2_S minerals before and after carbonation were tested, as shown in Figure 1b. It can be seen from Figure 1b that the asymmetric stretching vibration band (υ^3^) of the silicon–oxygen bond in the β-C_2_S mineral appears at 909 cm^−1^, indicating that the β-C_2_S mineral belongs to the nesosilicate structure with a bridging oxygen number of 0 (Q^0^). After carbonation curing, the υ^3^ of the silicon–oxygen bond shifts to a higher wavenumber (1085 cm^-1^, corresponding to Q^4^) [19,20], indicating that a highly polymerized SiO_2_ gel with a three-dimensional network structure was formed. Meanwhile, due to the reaction of the β-C_2_S mineral, the out-of-plane bending vibration (υ^4^) of the silicon–oxygen bond is also greatly weakened (located at 514 cm^−1^) after carbonation curing. In addition, after carbonation, the new absorption bands at 709, 876, and 1426 cm^−1^ are respectively the in-plane bending vibration (υ^2^), the out-of-plane bending vibration (υ^4^), and the asymmetric stretching (υ^3^) of carbon–oxygen bonds in CaCO_3_ crystal. Therefore, the carbonation reaction equation of the β-C_2_S mineral can be abbreviated as shown in Formula (1):

β-C_2_S + 2CO_2_ + H_2_O → 2CaCO_3_ + SiO_2_ (gels) + H_2_O (1)

### 3.2. Microstructure and Distribution of the Carbonation Products

**Figure 2:**
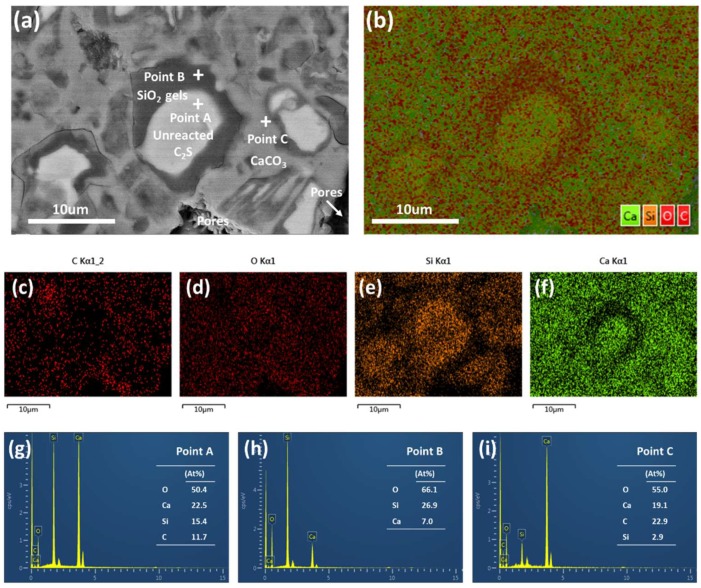
Backscattered electron (BSE) and energy-dispersive spectroscopy (EDS) images of β-C_2_S after carbonation. (**a**) BSE image; (**b**) elemental maps for composite element; (**c**–**f**) elemental maps for individual C, O, Si, and Ca, respectively; (**g**–**i**) EDS analysis of points A, B, and C.

Figure 2 shows a typical BSE image of the hardened β-C_2_S pastes after carbonation and the corresponding EDS spectrum. From Figure 2a, it was clearly observed that a dense microstructure was formed after carbonation. According to the grayscale features and the corresponding EDS maps (Figure 2g–i), the hardened β-C_2_S pastes were composed of pores (the darkest phase), SiO_2_ gel (average atomic number is 10.81, darker phase), CaCO_3_ (average atomic number is 12.56, gray phase), and uncarbonated C_2_S (average atomic number is 14.56, white phase). In addition, it can be seen from Figure 2a–f that the unreacted β-C_2_S particles are coated with a layer of SiO_2_ gel, and the pores between the original β-C_2_S particles are filled with CaCO_3_ crystals. The distribution of the carbonation products is closely related to the carbonation reaction process of β-C_2_S minerals.

### 3.3. Quantitative Analysis of the Carbonation Products

**Figure 3:**
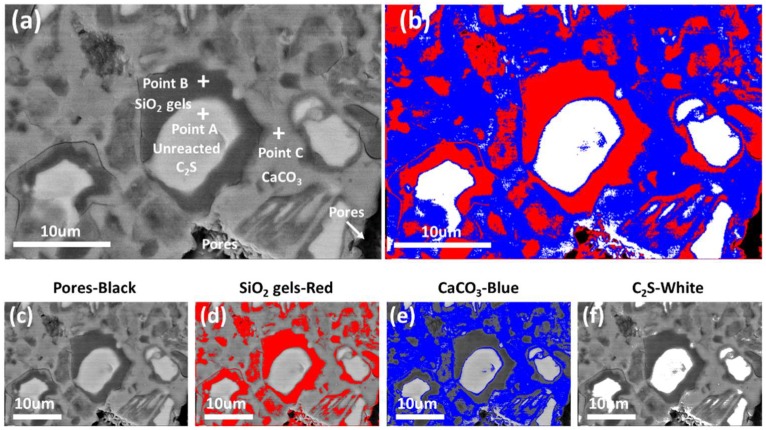
BSE images and phase distribution maps of β-C_2_S after carbonation. (**a**) BSE image; (**b**) images of the composite phases; (**c–f**) images of the individual phases after the gray level separation.

**Figure 4:**
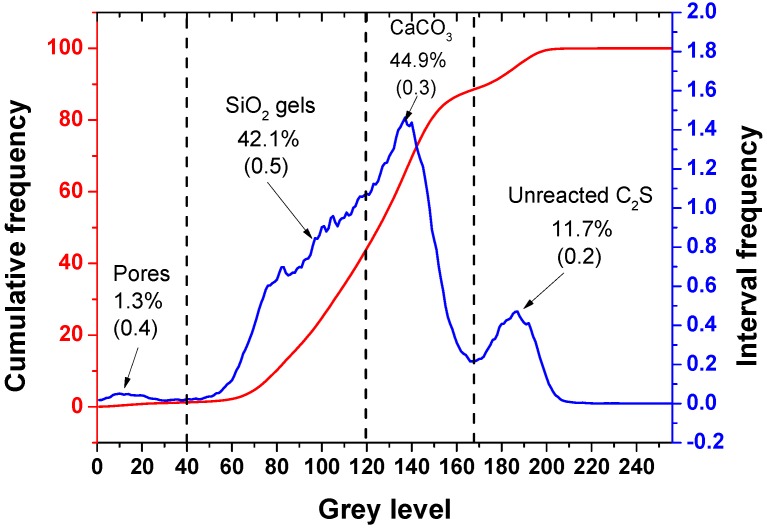
The gray level frequency and the cumulative distribution curves for different phases.

In order to further quantitatively analyze the relative volume fractions of each phase in the carbonation-hardened β-C_2_S pastes, the obtained BSE images were subjected to phase separation treatment using Image-Pro plus image analysis software. The phase distributions of the separated phases are shown in Figure 3c–f, representing pores, SiO_2_ gel, CaCO_3_, and uncarbonated β-C_2_S, respectively. The layered distributions of each phase of the hardened β-C_2_S pastes were clearly seen from Figure 3b. In addition, the gray level distribution histogram of different BSE images can be obtained by Image-Pro plus image analysis software. By counting the gray level distribution histograms of 10 BSE images, the gray level frequency and the cumulative distribution curves were obtained, as shown in Figure 4. The abscissa represents 0–255 gray levels, and the ordinate represents the frequency at which a certain gray level appears in the image. As can be seen from the gray level frequency distribution curve in Figure 4, there are four distinct gray level distribution intervals, (0–40), (40–120), (120–168), and (168–255), corresponding to pores, SiO_2_ gel, CaCO_3_, and uncarbonated β-C_2_S, respectively. According to the cumulative distribution curve, the relative volume fractions of the components were 1.3%, 42.1%, 44.9%, and 11.7%, respectively.

### 3.4. The Carbonation Hardening Mechanism of β-C_2_S Pastes

**Figure 5:**
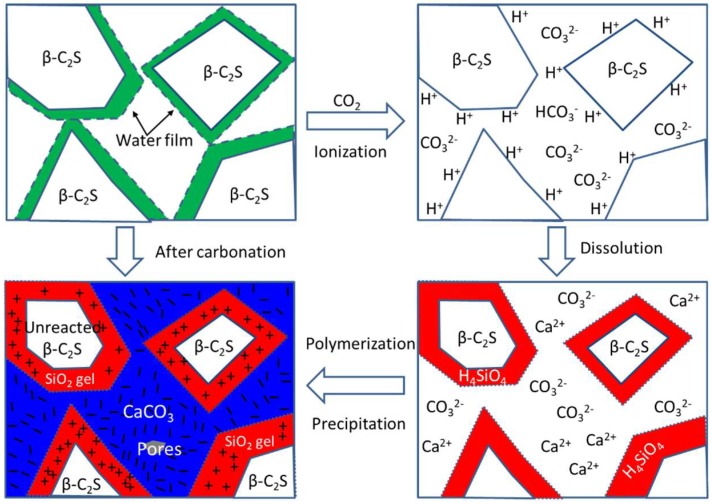
Schematic of carbonation hardening for β-C_2_S.

Based on the above results, the carbonation hardening mechanism of β-C_2_S pastes is shown in Figure 5. Before carbonation curing, β-C_2_S minerals are uniformly mixed with 10% of the mixing water, and then pressed and formed. The compacted β-C_2_S pastes are piled up by β-C_2_S mineral particles which are covered by a layer of water film. There are lots of pores between the particles. Since the hydration rate of β-C_2_S minerals is very slow, there is no adhesion between the particles. When the test block is placed into the carbonation reactor, the CO_2_ gas can rapidly diffuse into the compacted β-C_2_S pastes and dissolve in the water of the particle surface to form carbonic acid. The carbonic acid ionizes to produce H^+^, HCO_3_^−^, and CO_3_^2−^, and the reaction equation is as shown in the Formulas (2)–(4).

CO_2_ + H_2_O → H_2_CO_3_ (2)

H_2_CO_3_ → H^+^ + HCO_3_^−^ (3)

HCO_3_^−^ → H^+^ + CO_3_^2−^ (4)

It is generally believed that the hydration rate of β-C_2_S minerals is mainly controlled by the surface dissolution rate of β-C_2_S minerals. Under neutral conditions, β-C_2_S minerals dissolve slowly. However, under carbonation curing conditions, a large amount of H^+^ is generated due to the ionization of carbonic acid, and the pH of the pore solution is reduced from 7 to 4 at room temperature. Compared with neutral water, the elevated H^+^ concentration greatly accelerates the dissolution of Ca^2+^ and H_4_SiO_4_ from β-C_2_S minerals. Meanwhile, in a weakly acidic environment, H_4_SiO_4_ will gradually polymerize to form a three-dimensional network of SiO_2_ gel. Since H_4_SiO_4_ is more difficult to migrate than Ca^2+^, the resulting SiO_2_ gel is coated on the surface of the original β-C_2_S particles. The reaction equation is as shown in Formulas (5) and (6).

4H^+^ + 2CaO·SiO_2_ → 2Ca^2+^ + H_4_SiO_4_ (5)

H_4_SiO_4_ → SiO_2_ (gel) + 2H_2_O (6)

As the reaction proceeds, H^+^ is gradually consumed so that the ionization equilibrium of Formula (4) continues to the right. The resulting CO_3_^2−^ combines with the dissolved Ca^2+^ to precipitate in the pore solution to form CaCO_3_, as shown in Formula (7). Therefore, CaCO_3_ formed during the carbonation is filled between the pores of the original β-C_2_S particles.

Ca^2+^ + CO_3_^2−^ → CaCO_3_ (7)

With the continuous formation of SiO_2_ gel and CaCO_3_ crystal, the original loose β-C_2_S particles gradually bond to form a dense hardened structure. The formation of the hardened structure greatly hinders the diffusion rate of the reactants, and the carbonation reaction rate is greatly reduced, leaving an unreacted β-C_2_S center. Rapid generation of a sufficient number of SiO_2_ gels and CaCO_3_ crystals, and the formation of network structures of these carbonation products are responsible for the carbonation hardening of the β-C_2_S pastes.

## 4. Conclusions

In the present work, the composition and microstructure distributions of the carbonation products of hardened β-C_2_S paste were revealed by X-ray diffraction (XRD), Fourier transform-infrared (FT-IR) spectroscopy, and backscattered electron (BSE) image analysis. The main conclusions drawn are as follows:

1. After the carbonation curing of β-C_2_S for 24 h, a dense hardened paste was obtained. The hardened pastes are composed of pores, silica gel, calcium carbonate, and unreacted dicalcium silicate, with relative volume fractions of 1.3%, 42.1%, 44.9%, and 11.7%, respectively.
2. The unreacted β-C_2_S center is coated with a layer of SiO_2_ gel, and the pores between the original β-C_2_S particles are filled with CaCO_3_.
3. Rapid generation of a sufficient number of SiO_2_ gels and CaCO_3_ crystals, and the formation of network structures of these carbonation products are responsible for the carbonation hardening of the β-C_2_S mineral.
4. These results obtained may provide a profound understanding of the carbonation reaction process and hardening mechanism of low-calcium silicate-based cementitious materials.
